# Features of the Resonance in a Rectangular Dielectric Surace-Relief Gratings Illuminated with a Limited Cross Section Gaussian Beam

**DOI:** 10.3390/nano12010072

**Published:** 2021-12-28

**Authors:** Stefano Bellucci, Volodymyr Fitio, Iryna Yaremchuk, Oleksandr Vernyhor, Yaroslav Bobitski

**Affiliations:** 1 INFN-Laboratori Nazionali di Frascati, Via E. Fermi 54, 00044 Frascati, Italy; 2 Department of Photonics, Lviv Polytechnic National University, Bandera Street 12, 79013 Lviv, Ukraine; iryna.y.yaremchuk@lpnu.ua (I.Y.); vernyhor@gmail.com (O.V.); yaroslav.v.bobytskyi@lpnu.ua (Y.B.); 3 Department of Physics, University of Rzeszow, Pigonia Street 1, 35959 Rzeszow, Poland

**Keywords:** grating, waveguide, resonance, Gaussian beam, Fourier transform, one-dimensional photonic crystal

## Abstract

In this work the features of the resonance in a rectangular dielectric surface-relief gratings, illuminated with a limited cross-section Gaussian beam, have been studied. The rigorous coupled wave method and beam decomposition into the plane waves by the Fourier transform have been used. It is shown that there is a resonant wavelength for each thickness of the dielectric grating. The value of resonant wavelength depends on the beam angle of incidence on the gratings. Moreover, the two types of resonances can occur in the grating at certain grating parameters. The power reflection coefficient is practically equal to unity for the first type of resonance and is much smaller than unity, for the second one. The obtained results extend the knowledge regarding the nature of the waveguide resonance in the dielectric grating, considering the limited cross section beam, and they can increase its use in many applications.

## 1. Introduction

A waveguide coupling, filtering, focusing, field enhancement, nonlinear effects, and other effects can be obtained using waveguided mode resonant structures [1]. They are optical devices including planar waveguide with a periodic structure where incoming wave and output wave interfere in the waveguide [2]. Waveguide mode resonance occurs by the dielectric grating on the dielectric substrate under certain conditions. As a result, the reflection coefficient of such structure is equal to unity [3,4,5]. The resonance is disturbed when some parameters of the structure are changed. In particular, the reflection coefficient becomes smaller than unity when the refractive index of the surrounding medium is changed. Resonance is restored with a corresponding change in the incident light wavelength or with a suitable change in the angle of incidence. Therefore, such grating-based structures are used as the refractive index sensors of the liquid medium [6,7,8]. Several references related to the waveguide resonance by the grating can be found in the review [1]. A new method that can substantially improve hologram recording technology by eliminating complex processing procedures was also presented recently [9]. Relations between spectral and angular sensitivities for some types of sensors based on resonance phenomena are presented in [10]. A narrow-band optical filters can be synthesized, based on the dielectric grating on the dielectric substrate under guided mode resonance [11]. The two types of broadband and narrowband resonances can appear at the given structural parameters [12]. They are characterized by approximately the same spectral and angular sensitivities. However, there are significantly different full width at half maximum (FWHM) and, accordingly, different ratios FOM=S/FWHM (see Figure 5e,f in ref. [12]). Moreover, the electric fields arising in the grating in both types of resonance differ significantly in spatial distribution and magnitude (see Figure 5i,j in ref. [12]). Thus, the indicated features of resonances require additional research to explain the significant differences between the two types of resonance. It should be noted that gratings parameters and as a result their optical responses are also sensitive to the temperature changes [13,14,15]. It can be found a lot of works regarding optimization methods of the grating-based (photonic crystals) structures [16,17,18]. 

Numerical analysis of the plane wave diffraction by gratings is often carried out by rigorous coupled-wave analysis (RCWA) [19] using numerically stable S and R algorithms [20,21,22]. However, in practice, the gratings are illuminated with the limited cross-section beam. Particularly, it is a laser beam where the intensity distribution in the cross-section is described by the Gaussian function. The result of numerical analysis depends significantly on the beam cross-section size under resonance, as shown in [23,24]. Reflection coefficient of the resonant structure decreases and the FWHM increases when the beam width decreases. However, the angular and spectral sensitivities practically do not change [23]. The method described in [23] is based on the representation of limited beam in the form of the decomposition into the plane waves using the Fourier transform and RCWA [19,20] for calculations. The field distribution of the reflected and transmitted beams is determined by the inverse discrete Fourier transform. The number of plane waves in the numerical analysis must correspond to the sampling theorem [25]. This method is consistent with the energy conservation law for the dielectric grating [26].

It should be noted that a relatively small number of publications are devoted to the problem of limited cross section beam diffraction. It can be explained by the fact that the result of limited beam diffraction by the grating practically coincides with the result of plane wave diffraction at the resonance absence. The analysis of the diffraction of the limited size beam from 3 to 20 periods was presented in [27] using the finite-difference frequency-domain method. Diffraction efficiency slowly converges with the values assumed by RCWA, for the beam width of more than 20 periods. The influence of limited cross-section beams can be studied using the rigorous boundary element method [28]. Based on a simple scalar diffraction theory, it was shown that the angular anomaly of the reflected beam from the grating is a direct result of the beams finite size [29]. Theoretical modeling of the beam diffraction by the finite length metal grating under the surface plasmon-polariton resonances conditions shows the expansion of resonances when the grating size decreases. It is in a good fitting with experimental data [30]. Finite size gratings successfully used as a resonant filter for telecommunications due to the change in the angle of the incidence [31]. Moreover, fields of Gaussian beams scattered by reflective gratings differ markedly from those predicted by geometrical considerations using the angular spectral representation [32]. In addition, each diffracted beam has a lateral displacement. A corrected diffraction theory by the volume grating of the finite size, which is rather complicated for practical use, has been proposed in [33]. The influence of the incident Gaussian beam finite size on the anomalous reflection spectrum and the shape of the energy distribution in the reflected beam from the waveguide, with the grating, using the developed approximate theory, has been analyzed by the authors of [34]. An attempt to extend the RCWA method for the grating with the finite number of periods, using supercells, was made in the work [35]. Guizal et al. [36] developed a method called aperiodic RCWA. The dielectric constant of the finite grating is represented as Fourier integral. It leads to the integral-differential equation that can be solved using discretization in Fourier space. However, this method requires several hundred harmonics for convergence. Lalanne and co-authors [37,38] introduced the use of absorbing boundary conditions and perfect matching of layers at the ends of the unit cell, in order to numerically analyze finite periodic structures.

Therefore, using the finite diameter light beam diffraction on the grating [23] based on the RCWA method and the sampling theorem [25] will provide additional information regarding the nature of the resonance phenomena, in the dielectric grating system given in the work [12]. The RCWA is asymptotically accurate [20] and converges faster than the other methods for dielectric periodic structures.

## 2. Theoretical Backgrounds of the Limited Cross-Section Beam Diffraction Analyzing Method

**Figure 1:**
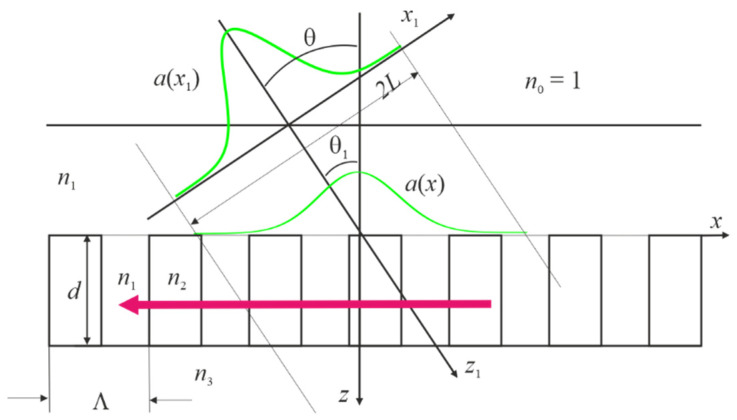
Incidence of the Gaussian beam on the grating. The grating parameters are as follows: Λ = 0.70 μm, *n*_1_ = 1.33, *n*_2_ = 2, *n*_3_ = 1.45, *F* = 0.5. The angle *θ* takes only two values, i.e., 0 and *π*/10. The wavelength *λ* and the grating thickness *λ* are variables. Red arrow represents waveguide mode propagation.

Let us consider the case when the beam propagates in air at the angle θ and it is incident on the infinite grating at the angle $\theta_{1}$ in the medium with the refractive index $n_{1}$ (Figure 1). Grating parameters were selected based on the result of several our previous numerical experiments [10,22,23]. Moreover, structures with such parameters can be fabricated experimentally. It should be noted that actual waveguide resonance-based volume phase gratings can be fabricated by the holographic recording using the symmetric two-beam setup for the fabrication of transmission gratings. The detailed description of manufacturing process is presented in [8]. The high uniformity in thickness and accurate reproduction of the periodicity allows us to expect a good fitting between the results of numerical modeling and experimental data.

The distribution of the amplitude along x coordinate is described by the function a(x). The function a(x) is practically zero outside the interval $\left[-x_{max}/2,\;x_{max}/2\right]$.

Let us perform the Fourier transform of the function a(x).

(1) $$A\left(u\right)=\int_{-\infty }^{\infty }a\left(x\right)\exp\left(i2\pi u_{0}x\right)\exp\left(-i2\pi ux\right)dx,$$

where $u_{0}=\frac{n_{1\;}}{\lambda }\sin\theta_{1}$.

If $A_{0}\left(u\right)=\int_{-\infty }^{\infty }a\left(x\right)\exp\left(-i2\pi ux\right)dx,$ then

(2) $$A\left(u\right)=A_{0}\left(u-u_{0}\right),$$

according to the Fourier shift theorem [25]. 

We can determine $a\left(x\right)\exp\left(i2\pi u_{0}x\right)$ using the inverse Fourier transform, knowing A(u), as follows:

(3) $$a\left(x\right)\exp\left(i2\pi u_{0}x\right)=\int_{-\infty }^{\infty }A\left(u\right)\exp\left(i2\pi ux\right)du.$$

The function A(u) is practically equal to zero outside the interval $\left[u_{0}-u_{max}/2,\;u_{0}+u_{max}/2\right]$. The continuous frequency range $\left[u_{0}-u_{max}/2,\;u_{0}+u_{max}/2\right]$ is discretized by dividing into M − 1 interval as:

(4) $$u_{m}=u_{0}-\frac{M+1}{2}\delta u+m\delta u=\frac{n_{1}}{\lambda }\sin\theta_{m},$$

where $\delta u=\frac{u_{max}}{M-1}$, m is an integer number which ranges from 1 to M. It is more convenient if M is an odd integer. In accordance with the sampling theorem [25], to pass from continuous coordinates and frequencies to discrete ones, and to use the discrete Fourier transform, the following condition must be satisfied:

(5) $$x_{max}u_{max}=M-1\gg 1,$$

The discrete coordinates can be expressed as follows:

(6) $$x_{m}=-\frac{x_{max}}{2}+\left(m-1\right)\frac{x_{max}}{M-1}=-\frac{x_{max}}{2}+\left(m-1\right)\delta x.$$

The spatial frequency $u_{max}$ must satisfy the Parseval equality condition [25]:

(7) $$\overset{u_{0}+u_{max}/2}{\underset{u_{0}-u_{max}/2}{\int }}{\left|A\left(u\right)\right|}^{2}du\approx \overset{\infty }{\underset{-\infty }{\int }}{\left|a\left(x\right)\right|}^{2}dx.$$

The right-hand side of Equation (7) is proportional to the power of the incident beam. Moreover, the energy Equation (7) is the criterion for choosing the frequency $u_{max}.$ The value of the left-hand side of Equation (7) will tend to the value of the right part, when $u_{max}$ increases. According to the following ratio, δu and M can be chosen as $\sum_{j=-\frac{M-1}{2}}^{\frac{M-1}{2}}{\left|A\left(j\delta u\right)\right|}^{2}\delta u\approx \int_{-\infty }^{\infty }{\left|a\left(x\right)\right|}^{2}dx,$ where $\delta u=\frac{u_{max}}{M-1}.$ The value of the left part of this ratio tends to the value of the right part, with the decrease in δu due to the increase in M. In addition, the relations 1δx≥umax, 1δu≥xmax [25] must be satisfied. In our analysis, it is assumed that 1δx=umax, 1δu=xmax. The beam with the spatial distribution of the amplitude $a\left(x\right)\exp\left(i2\pi u_{0}x\right)$ can be represented as a set of plane waves with the amplitude $A\left(u_{m}\right)\delta u$. Each plane wave with number *m* is incident on the grating at the angle $\theta_{m}$. Thus, it can be written as follows:

(8) $$a\left(x\right)\exp\left(i2\pi u_{0}x\right)\approx \overset{M}{\underset{m=1}{\sum }}A\left(u_{m}\right)\exp\left(i2\pi u_{m}x\right)\delta u.$$

The result of the diffraction of each plane wave with amplitude $A\left(u_{m}\right)\delta u$ and number *m* can be calculated using the RCWA method. The amplitudes of the reflected and transmitted waves, which are denoted by $r_{0m}$ and $t_{0m}$ for the zero order, will be obtained. In this case, for convenience, $r_{0m}$ and $t_{0m}$ are calculated at the unit amplitude of the incident wave. Thus, the total amplitudes of the reflected $r_{0}\left(x\right)$ and transmitted waves $t_{0}\left(x\right)$ at the medium/grating interface can be obtained using the discrete inverse Fourier transform as follows:

(9) $$r_{0}\left(x\right)=\overset{M}{\underset{m=1}{\sum }}A\left(u_{m}\right)r_{0m}\left(u_{m}\right)\exp\left(i2\pi u_{m}x\right)\delta u,$$

(10) $$t_{0}\left(x\right)=\overset{M}{\underset{m=1}{\sum }}A\left(u_{m}\right)t_{0m}\left(u_{m}\right)\exp\left(i2\pi u_{m}x\right)\delta u.$$

It is possible to calculate quantities proportional to the power distributions of the reflected wave along the grating and the wave transmitted through the gratings, based on Equations (9) and (10), in accordance with the expressions:

(11) $$R_{0}\left(x\right)={\left|r_{0}\left(x\right)\right|}^{2}n_{1}\cos\theta_{1},\;T_{0}\left(x\right)={\left|t_{0}\left(x\right)\right|}^{2}n_{3}\cos\theta_{3}.$$

The relative powers of reflection and transmission of the grating can be expressed as follows:

(12) $$P_{r}=\frac{\int_{-x_{max}/2}^{x_{max}/2}R_{0}\left(x\right)dx}{\cos\theta_{1}n_{1}\int_{-\infty }^{\infty }{\left|a\left(x\right)\right|}^{2}dx},\;P_{t}=\frac{\int_{-x_{max}/2}^{x_{max}/2}T_{0}\left(x\right)dx}{n_{1}\cos\theta_{1}\int_{-\infty }^{\infty }{\left|a\left(x\right)\right|}^{2}dx}.$$

Evidently, the condition $P_{r}$ + $P_{t}\approx$ 1 corresponding to the energy conservation law at such definition of $P_{r}$ and $P_{t}$ in the absence of absorption in the structure must be satisfied.

## 3. Results

All numerical results were obtained using 41 coupled waves in the RCWA method for Transverse Electric (TE) waves. The increase in the number of coupled waves led to a small change in the reflection and transmission coefficients of the plane wave and the Gaussian beam, which are not separated in the figures. The refractive indices of the periodic structure components are constant and their data are given in the caption of Figure 1.

**Figure 2:**
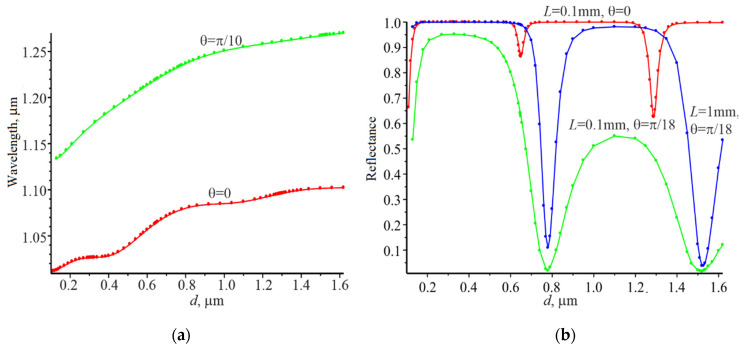
Dependence of the resonant wavelength on the grating thickness for the plane wave(**a**); dependence of the relative reflection power *P_r_* on the grating thickness for the Gaussian beam (**b**), *L* is the full width at half maximum of the Gaussian beam in accordance with Figure 1. Gaussian beam expansion in 1001 plane waves was used for calculations.

Firstly, the resonance wavelengths for the plane wave incidence on the grating were determined for a series of thicknesses d that are in the wavelength range from 0.13 to 1.62 μm. The resonant wavelengths are selected with the accuracy at which the reflection coefficient of the grating is higher than 0.99 in the zero-diffraction order for the selected thicknesses. The resonant wavelength for which the reflection coefficient is equal to unity can be chosen for a non-absorbing structure and for each grating thickness. Corresponding dependencies are shown in Figure 2a. The red curve is obtained at normal incidence and the green one at the incidence of the plane wave at the angle θ=π/10. Dependences of the relative reflection power $P_{r}$ of the grating for the Gaussian beam at the normal incidence (red curve) and at the angle incidence θ=π/10 (green and blue curves) are presented in Figure 2b. Resonant wavelength increases with increasing grating thickness. In addition, the resonant wavelengths at the angle θ=π/10 plane wave incidence are higher than at the normal plane wave incidence for the same grating thickness.

Reflection coefficients $P_{r}$ for the Gaussian beam depending on the grating thickness at the corresponding resonant wavelength according to Figure 2a are shown in Figure 2b. The red curve represents the normal incidence of the Gaussian beam with the width of L=0.1 mm. The reflection coefficient is close to unity in the whole wavelength range (grating thicknesses) except for the narrow spectral bands which correspond to the narrow range of grating thicknesses. The first reflection minimum ($P_{r}=0.87$) is observed at the wavelength of 1.06 μm and the grating thickness of 0.65 μm. The second reflection minimum ($P_{r}=0.62$) was obtained at the wavelength of 1.09 μm and the grating thickness of 1.30 μm. The blue and green curves are obtained at L=1 mm and L=0.1 mm, respectively, when the Gaussian beam is incident at the angle θ=π/10. The minima for both curves coincide in the thickness of the grating and in the corresponding wavelength. Reflection coefficients are 0.11 (blue curve) and 0.02 (green curve), respectively, for the thickness of d=0.78 μm and the wavelength of 1.20 μm. Reflection coefficients are 0.04 (blue curve) and 0.02 (green curve) for higher grating thickness d=1.52 μm and for the corresponding resonance wavelength 1.27 μm. The minima of the reflection coefficient are much lower at the angle of incidence θ=π/10 than at the normal incidence of the Gaussian beam. It should be noted that the reflection in the high-reflectance region is close to unity for the blue curve (L=1 mm).

This feature of the reflection coefficient dependence on the grating thickness at the resonant wavelengths can be explained as follows. The average refractive index of the grating $\bar{n}=\left[n_{1}\left(1-F\right)+n_{2}F\right]/2$ is higher than $n_{1}$ and $n_{3}$. Therefore, the grating can be works like a waveguide and the high reflection coefficient is possible when waveguide resonance occurs [3,7,9]. On the other hand, the grating can be represented as a one-dimensional photonic crystal with forbidden and allowed band gaps. In the considered grating, the periodical change in the refractive index has a high contrast, respectively, $n_{1}=1.33$ and $n_{2}=2$. Therefore, if such grating is considered as a 1D photonic crystal, then such crystal can have both allowed and forbidden band gaps [39]. Currently, there is no exact theory to find the boundaries between the forbidden and allowed photonic band gaps for the grating. The theory for the 1D photonic crystal of the interference mirror type can be used. However, this method will be approximate and will agree with Figure 2 for higher thicknesses. In our case the method of pointer function [40] has been used to find the boundaries between the photonics band gaps. If the wavelength is in the forbidden zone when the Gaussian beam is incident on the grating, then the waveguide mode will propagate over a short distance. The reflection of the grating will be high, due to the strong interaction of the waveguide mode with the grating. The waveguide mode will propagate over a large distance in the grating if wave is in the allowed band. The interaction of the waveguide mode with the grating will be weak, leading to the low reflection of the grating. The following numerical analysis results support this qualitative analysis.

**Figure 3:**
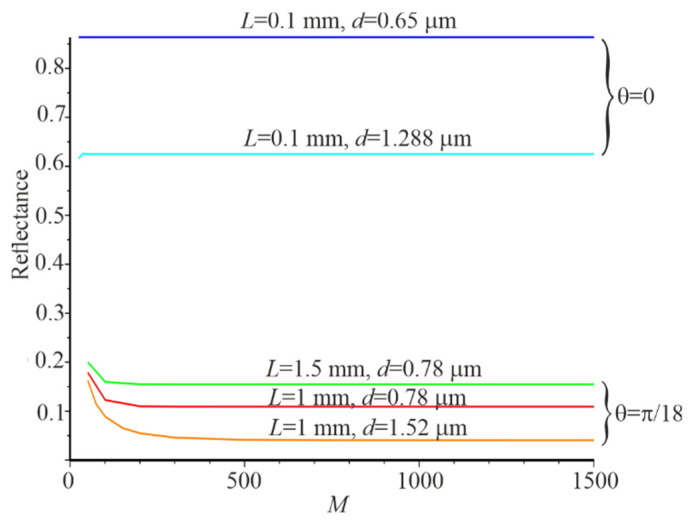
Dependences *P_r_* of the plane waves used in the Gaussian beam expansion under different resonance conditions.

The dependence of the reflection $P_{r}$ on the number of plane waves M has been calculated for several resonance conditions. It was done to determine the number of plane waves used in the Gaussian beam expansion. Results of numerical simulation will be close to the asymptotic values using this data. Corresponding dependencies are shown in Figure 3. $P_{r}$ for all resonance conditions is close to the asymptotic value at M=1001. The lower $P_{r}$, the slower $P_{r}$ tends to the boundary value. This is especially evident when the angle of Gaussian beam incidence is θ=π/18. Therefore, all our calculations were performed at M=1001.

**Figure 4:**
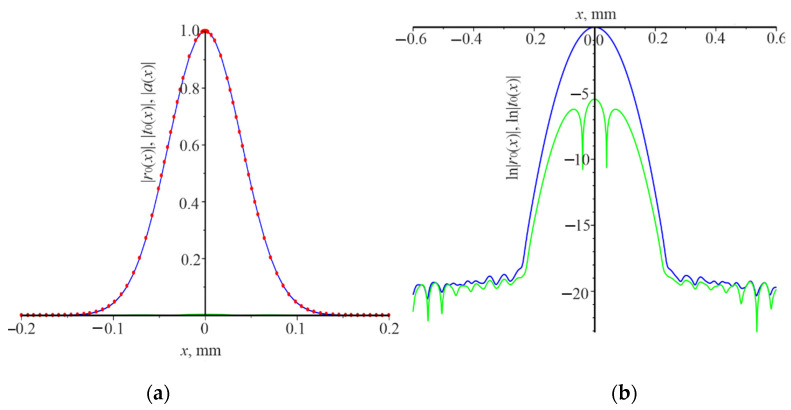
The distribution of the field amplitude along the *x* coordinate of the incident Gaussian beam |*a*(*x*)| (red dots), reflected beam |*r*_0_(*x*)| (blue curve), the transmitted beam |*t*_0_(*x*)| (green curve) (**a**). ln|*r*_0_(*x*)| (blue curve), ln|*t*_0_(*x*)| (green curve) (**b**). Numerical calculations were carried out under the conditions *d* = 0.32 μm, *λ* = 1.03 μm, *θ* = 0, *L* = 0.1 mm.

The distribution of the field amplitudes moduli of the reflected $\left|r_{0}\left(x\right)\right|$ and the transmitted beam $\left|t_{0}\left(x\right)\right|$ can give a deeper understanding of the Gaussian beam diffraction features. It is, for the natural logarithms of these quantities under different resonance conditions. The corresponding dependences for the resonant grating thickness d=0.32 μm and the resonant wavelength λ=1.03 μm, the angle θ=0, and L=0.1 mm are presented in Figure 4. The Gaussian beam is normally incident on the grating. The power reflection coefficient $P_{r}$ is equal to 0.99. Distribution of the incident beam amplitude modulus |a(x)| (red dots) coincides with the distribution of the reflected beam amplitude modulus |a(x)| (blue curve). The amplitude modulus of the transmitted beam $\left|t_{0}\left(x\right)\right|$ (green curve) is practically zero. If grating is considered as the photonic crystal, then the waveguide mode propagates in the grating as in the waveguide. It is in the photonic band gap and rapidly decays during the propagation in the grating, due to the matched phase reflection from each period (like in an interference mirror).

There is a strong interaction of the waveguide mode with the grating. As a result, the reflection coefficient of the grating $P_{r}$ is practically equal to unity. It contributes to the rapid decay of the waveguide mode in the grating. It should be noted that the strong interaction of the waveguide mode with the grating is not enough to obtain the high reflection coefficient. There, another resonance occurs leading to the increase in the field of the waveguide mode. The effective cavity length is slightly higher than the Gaussian beam width. Therefore, the resonance ($P_{r}\approx 1$) occurs at the certain wavelength for each grating thickness, as shown in Figure 2a. Specially features are not observed in Figure 4b, except that $\left|t_{0}\left(x\right)\right|$ and $\left|r_{0}\left(x\right)\right|$ practically decrease to digital noise at x=±0.3 mm.

**Figure 5:**
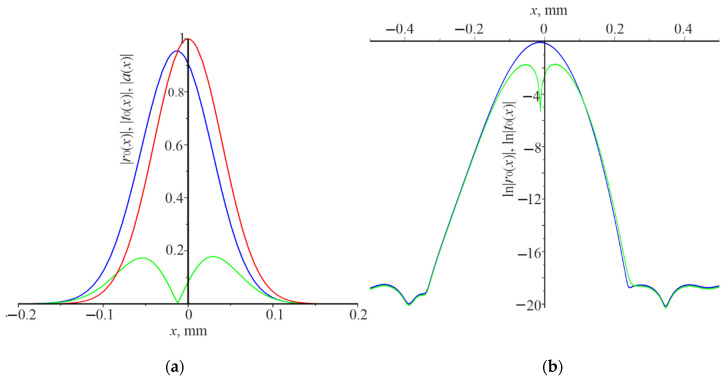
The distribution of the field amplitude along the *x* coordinate of the incident Gaussian beam |*a*(*x*)| (red curve), reflected beam |*r*_0_(*x*)| (blue curve), the transmitted beam |*t*_0_(*x*)| (green curve) (**a**). ln|*r*_0_(*x*)| (blue curve), ln|*t*_0_(*x*)| (green curve) (**b**). Numerical calculations were carried out under the conditions *d* = 0.32 μm, *λ* = 1.17 μm, *θ* = *π*/18, *L* = 0.1 mm.

The distribution of moduli amplitudes of the reflected field $\left|r_{0}\left(x\right)\right|$ and the transmitted beam $\left|t_{0}\left(x\right)\right|$, as well as the natural logarithms of these quantities under the following resonance conditions d=0.32 μm, λ=1.17 μm, L=0.1 mm are presented in Figure 5. The Gaussian beam angle incidence is π/18. The power reflection coefficient $P_{r}$ is about 0.95. The field amplitude distribution in the reflected beam is shifted to the left by hundredths of a millimeter, relative to the incident beam, and is similar in shape to the incident beam.

There is the overlap of curves for the reflected and transmitted beams, as well as a small linear section in the interval [−0.35 mm,−0.25 mm], for logarithmic curves at approximately x<−0.2 mm. The shape of the logarithmic curves for x>0 differs from the curves for x<0. Particularly, there is no linear section in the dependencies of reflection and transmission, and there is also no overlap. The waveguide mode decays very fast, during the propagation in the grating. It is due to the strong interaction of the grating and light emission at the zero order. In terms of the photonic crystal, the waveguide mode is in the band gap. Reflection from the grating grooves is phased and the resonator is somewhat larger than 2L.

**Figure 6:**
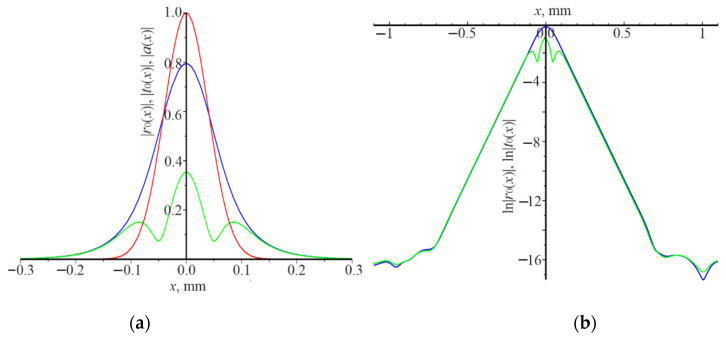
The distribution of the field amplitude along the *x* coordinate of the incident Gaussian beam |*a*(*x*)| (red curve), reflected beam |*r*_0_(*x*)| (blue curve), the transmitted beam |*t*_0_(*x*)| (green curve) (**a**); ln|*r*_0_(*x*)| (blue curve), ln|*t*_0_(*x*)| (green curve) (**b**). Numerical calculations were carried out under the conditions *d* = 0.65 μm, *λ* = 1.07 μm, *θ* = 0, *L* = 0.1 mm.

The distribution of moduli amplitudes of the reflected field $\left|r_{0}\left(x\right)\right|$ and the transmitted beam $\left|t_{0}\left(x\right)\right|,$ as well as the natural logarithms of these quantities under the following resonance conditions: d=0.65 μm, λ=1.07 μm, L=0.1 mm, are presented in Figure 6. The Gaussian beam is normally incident on the grating. The power reflection coefficient $P_{r}$ is 0.87. There is the extension $\left|r_{0}\left(x\right)\right|$ and $\left|t_{0}\left(x\right)\right|$ compared with the beam width |a(x)|. Moreover, $\left|r_{0}\left(x\right)\right|$ and $\left|t_{0}\left(x\right)\right|$ practically coincide for |x|>0.15 (Figure 6a). In addition, $\ln\left|r_{0}\left(x\right)\right|$ and $\ln\left|t_{0}\left(x\right)\right|$ decrease linearly for |x|>0.15, that is $\left|r_{0}\left(\pm x\right)\right|=\left|r_{0}\left(\pm 0.15\right)\right|\exp$(−γ|x|), where γ= 22.02 mm^−1^.

It should be noted that γ is independent of the beam width. In this case, the waveguide mode is in the allowed photonic band gap from the point of view of the theory of the photonic crystal. It is demonstrated by the strict linearity and coincidence of $\ln\left|r_{0}\left(x\right)\right|$ and $\ln\left|r_{0}\left(x\right)\right|$ for |x|>0.15. Therefore, it propagates over the long distance, compared to the width of the incident Gaussian beam. At the same time, there is the strong interaction of the waveguide mode with the grating, which provides a high reflection ($P_{r}=$ 0.873).

**Figure 7:**
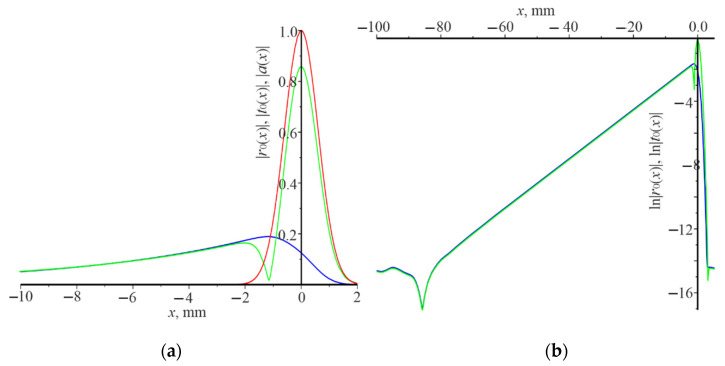
The distribution of the field amplitude along the *x* coordinate of the incident Gaussian beam |*a*(*x*)| (red curve), reflected beam |*r*_0_(*x*)| (blue curve), the transmitted beam |*t*_0_(*x*)| (green curve) (**a**); ln|*r*_0_(*x*)| (blue curve), ln|*t*_0_(*x*)| (green curve) (**b**). Numerical calculations were carried out under the conditions *d* = 0.78 μm, *λ* = 1.23 μm, *θ* = *π*/18, *L* = 1.5 mm.

The distribution of moduli amplitudes of the reflected $\left|r_{0}\left(x\right)\right|$ and the transmitted beams $\left|t_{0}\left(x\right)\right|$, as well as the natural logarithms of these quantities under the following resonance conditions d=0.78 μm, λ=1.23 μm, L=1.5 mm, are presented in Figure 7. The angle Gaussian beam incidence is θ=π/18. It was taken that L=1.5 mm for numerical calculations, to obtain the not too small value of the power reflection coefficient $P_{r}$. It is equal to 0.155 under these conditions. The waveguide mode is formed due to the resonant interaction of the Gaussian beam with the grating. It is in the allowed photonic band gap. The waveguide mode decays very slowly during the propagation in the grating due to weak interaction with the grating and light emission at the zero order. These features are demonstrated in Figure 7. It is possible to determine the attenuation decrement of the waveguide mode γ using the linear section of $\ln\left|r_{0}\left(x\right)\right|$ (Figure 2b), which is $0.154\;{\mathrm{mm}}^{-1}$ in this case.

**Figure 8:**
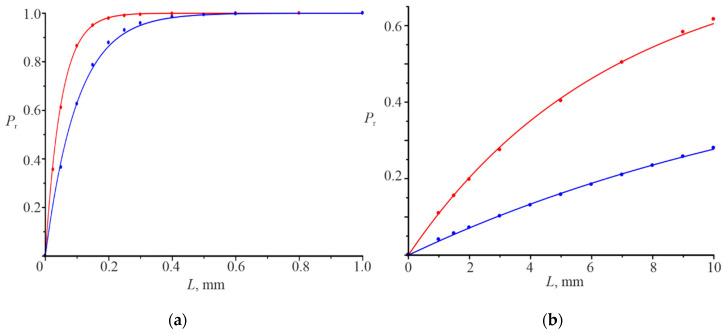
Dependencies of the reflection coefficient *P_r_* on the Gaussian beam width at *d* = 0.65 μm, *λ* = 1.06 μm, *θ* = 0, *γ* = 22.02 mm^−1^ (red curve), *d* = 1.30 μm, *λ* = 1.09 μm, *θ* = 0, *γ* = 11.1 mm^−1^ (blue curve) (**a**); *d* = 0.78 μm, *λ* = 1.23 μm, *θ* = *π*/18, *γ* = 0.15 mm^−1^ (red curve), *d* = 1.52 μm, *λ* = 1.27 μm, *θ* = *π*/18, *γ* = 0.05 mm^−1^ (blue curve) (**b**).

Results of calculations of the reflection coefficient $P_{r}$ dependences on the Gaussian beam width are shown in Figure 8. The points correspond to the results of numerical simulation. Continuous curves are described by the corresponding approximation equations. Calculation presented in Figure 8a were carried out for the normal incidence of the Gaussian beam and in Figure 8b at the angle of incidence θ=π/18. All these dependences were calculated at the linear dependences of $\ln\left|r_{0}\left(x\right)\right|$ and $\ln\left|t_{0}\left(x\right)\right|$ at significant intervals of change x. Linear sections $\ln\left|r_{0}\left(x\right)\right|$ and $\ln\left|t_{0}\left(x\right)\right|$ are characterized by certain damping decrements.

The curves in Figure 8a are approximatively described by simple approximation expressions in which the damping decrement γ is present as follows:

(13) $$P_{r}\left(L\right)\approx 1-\exp\left(-0.9\gamma L\right).$$

There γ is equal $22.02\;{\mathrm{mm}}^{-1}$ and are $11.1\;{\mathrm{mm}}^{-1}$, respectively, for the red and blue curves. Therefore, it is the same Equation (13) for both curves shown in Figure 8a. The curves in Figure 8b are described by more complex approximation expressions and slightly differing from each other, but there is also the damping decrement.

The red curve can be approximated with the following equation:

(14) $$P_{r}\left(L\right)\approx 1-\exp\left[-\gamma \left(0.775L-0.016L^{2}\right)\right],\;\gamma =0.1538\;{\mathrm{mm}}^{-1}.$$

The blue curve is described by the next equation:

(15) $$P_{r}\left(L\right)\approx 1-\exp\left[-\gamma \left(0.775L-0.01L^{2}\right)\right],\;\gamma =0.0473{\;\mathrm{mm}}^{-1}$$

The value γ has the main influence on the dependence $P_{r}\left(L\right)$ (on the rate of its change on L) and $P_{r}\left(L\right)$ tends to unity with increasing L. It is possible to calculate the power transmission $P_{t}\left(L\right)$ considering that $P_{r}\left(L\right)+$ $P_{t}\left(L\right)=1,$ based on Equations (13)–(15) for the dielectric gratings.

## 4. Discussions

Results show that power reflection coefficient can vary over a wide range (practically from unity to zero), depending on the resonant grating thickness, when the Gaussian beam is incident on gratings under resonance (see Figure 2b). We have $P_{r}=0.99$ even for the Gaussian beam full half-width at the maximum L=0.1 mm for the grating thickness of 0.32 μm at normal incidence and $P_{r}=0.87$ for the grating thickness of 0.65 μm. If the angle of Gaussian beam incidence (L=1 mm) becomes equal to π/18, then $P_{r}=0.99$ at the thickness d=0.32 μm. The power reflection coefficient will become equal to 0.11 at the thickness d=0.78 μm.

This difference in power reflection coefficient can be explained as follows. The grating operates as the waveguide, when the average refractive index of the grating $\bar{n}=\left[n_{1}\left(1-F\right)+n_{2}F\right]/2$ is higher than $n_{1}$ and $n_{2}$. The waveguide mode is excited in the grating when the plane wave is incident under resonance. On the other hand, such a grating is a one-dimensional photonic crystal, which has allowed and forbidden photonic band gaps. If the waveguide mode, excited by the incident Gaussian beam, is in the forbidden photonic band gap, then it cannot propagate over a considerable distance and changes its propagation to the opposite one (a similar situation arises in the interference mirror). In fact, the waveguide mode is in a resonator trap, the length of which is somewhat longer than 2L. Two waveguide modes form a standing wave since they are excited at the normal incidence. The waveguide modes interact with the grating during propagation. Consequently, the energy of the modes is converted into the energy of the reflected and transmitted beams. This case is shown in Figure 4a, where resonance occurs under the following conditions: d=0.32 μm, λ=1.03 μm, θ=0, L=0.1 mm. The amplitude distribution of the reflected beam $\left|r_{0}\left(x\right)\right|$ coincides with the amplitude distribution of the incident beam |a(x)|. The amplitude of the transmitted beam is practically zero. In Figure 4b there are no linear sections of the change $\ln\left|r_{0}\left(x\right)\right|$ and $\ln\left|t_{0}\left(x\right)\right|$. Thus, there it is impossible to determine the damping decrement γ of the waveguide modes during propagation in the grating. Figure 5 demonstrates a slightly different situation, where resonance occurs, under the following conditions: d=0.32 μm, λ=1.17 μm, θ=π/18, L=0.1 mm. One waveguide mode is excited at the wavelength λ=1.17 μm which propagates from right to left since the angle of incidence is different from zero. There is some displacement of the reflected and transmitted beams to the left, relative to the incident beam on the grating, and their widths are somewhat longer than 2L. The linear section of the change $\ln\left|r_{0}\left(x\right)\right|$ and l $\ln\left|t_{0}\left(x\right)\right|$ is negligible (see Figure 5b). Therefore, it can be argued that in this case the waveguide mode also is in the forbidden photonic band gap. Another case is realized in Figure 6 (normal incidence of the Gaussian beam on the grating) and Figure 7 (incidence of the Gaussian beam at the angle of π/18), when the waveguide modes are in the allowed photonic band gaps. The reflected and transmitted beams are much wider than the incident beam. Their width is determined by the power of interaction of the waveguide mode with the grating. The length of the linear dependence of the logarithms $\ln\left|r_{0}\left(x\right)\right|$ and $\ln\left|t_{0}\left(x\right)\right|$, respectively. γ, as well as the power reflection coefficient $P_{r}$, depend on the power of interaction.

The dependence of the power reflection coefficient $P_{r}$ on the Gaussian beam width $P_{r}$ is determined by the damping decrement and increases to unity with the increase in L.

## 5. Conclusions

The diffraction of the surface-relief gratings illuminated with a limited cross section of the Gaussian Beam has been studied by the method based on RCWA and the decomposition of the plane waves using the Fourier transform. It is shown that there is the resonant wavelength for each dielectric grating thickness, the value of which depends on the beam angle of incidence on the grating. The power reflection coefficient changes practically from unit to zero, depending on the resonant grating thickness at the resonant Gaussian beam incidence. This difference in the power reflection coefficient can be explained by the fact that the waveguide mode, excited by the incident Gaussian beam, is in the forbidden photonic band gap. It cannot propagate over a considerable distance and changes its propagation to the opposite one. It was shown that it is impossible to determine the damping decrement γ of the waveguide modes during the propagation in the grating at certain grating parameters. In addition, the damping decrement and the power reflection coefficient depend on the power of the waveguide mode-grating interaction. In general, the results of this work will be useful in the development of refractive index sensors since the principle of operation of the sensors is based on the waveguide resonance in dielectric gratings. Moreover, the sensitivity of resonance-based sensors is much higher in comparison to, for example, the holographic sensors.

## Data Availability

The data presented in this study are available on reasonable request from the corresponding author.
